# Hexakis(2-amino­pyridinium) di-μ_6_-oxido-tetra-μ_3_-oxido-tetra­deca-μ_2_-oxido-octa­oxidodeca­vanadium(V) dihydrate

**DOI:** 10.1107/S1600536809004334

**Published:** 2009-02-13

**Authors:** Caixia Yuan, Liping Lu, Miaoli Zhu, Qi Ma, Yanbo Wu

**Affiliations:** a Institute of Molecular Science, Key Laboratory of Chemical Biology and Molecular Engineering of the Education Ministry, Shanxi University, Taiyuan, Shanxi 030006, People’s Republic of China

## Abstract

In the title compound, (C_5_H_7_N_2_)_6_[V_10_O_28_]·2H_2_O, the [V_10_O_28_]^6−^ anion is generated by crystallographic inversion symmetry and each of the five vanadium centres adopts a distorted VO_6_ octa­hedral geometry. In the crystal structure, a network of N—H⋯O, N—H⋯(O,O) and O—H⋯O hydrogen bonds helps to establish the packing.

## Related literature

For a related structure, see: Gong *et al.* (2006 ▶). For background to the biological activity of oxovanadates and peroxovanadium compounds, see: Pacigová *et al.* (2007 ▶).
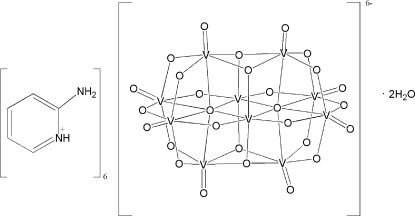


## Experimental

### 

#### Crystal data

(C_5_H_7_N_2_)_6_[V_10_O_28_]·2H_2_O
*M* *_r_* = 1564.19
Monoclinic, 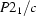

*a* = 9.840 (3) Å
*b* = 18.180 (6) Å
*c* = 14.299 (5) Å
β = 97.416 (4)°
*V* = 2536.6 (14) Å^3^
*Z* = 2
Mo *K*α radiation
μ = 1.86 mm^−1^
*T* = 298 (2) K
0.40 × 0.20 × 0.20 mm

#### Data collection

Bruker SMART 1K CCD diffractometer
Absorption correction: multi-scan (*SADABS*; Bruker, 2000 ▶) *T*_min_ = 0.523, *T*_max_ = 0.707
9858 measured reflections
4341 independent reflections
3949 reflections with *I* > 2σ(*I*)
*R*_int_ = 0.042

#### Refinement

*R*[*F* ^2^ > 2σ(*F* ^2^)] = 0.074
*wR*(*F* ^2^) = 0.128
*S* = 1.29
4341 reflections
370 parameters
H-atom parameters constrained
Δρ_max_ = 0.53 e Å^−3^
Δρ_min_ = −0.45 e Å^−3^

### 

Data collection: *SMART* (Bruker, 2000 ▶); cell refinement: *SAINT* (Bruker, 2000 ▶); data reduction: *SAINT*; program(s) used to solve structure: *SHELXS97* (Sheldrick, 2008 ▶); program(s) used to refine structure: *SHELXL97* (Sheldrick, 2008 ▶); molecular graphics: *SHELXTL/PC* (Sheldrick, 2008 ▶); software used to prepare material for publication: *SHELXTL/PC*.

## Supplementary Material

Crystal structure: contains datablocks I, global. DOI: 10.1107/S1600536809004334/hb2897sup1.cif

Structure factors: contains datablocks I. DOI: 10.1107/S1600536809004334/hb2897Isup2.hkl

Additional supplementary materials:  crystallographic information; 3D view; checkCIF report

## Figures and Tables

**Table 1:** Selected bond lengths (Å)

V1—O7	1.616 (4)
V1—O3	1.767 (4)
V1—O8	1.842 (4)
V1—O2	1.991 (4)
V1—O12	2.038 (4)
V1—O1	2.250 (4)
V2—O9	1.608 (4)
V2—O6	1.794 (4)
V2—O10	1.823 (4)
V2—O12^i^	2.015 (4)
V2—O2^i^	2.017 (4)
V2—O1	2.243 (4)
V3—O11	1.594 (4)
V3—O14	1.827 (4)
V3—O8	1.871 (4)
V3—O6	1.891 (4)
V3—O5	2.045 (4)
V3—O1	2.305 (4)
V4—O4	1.676 (4)
V4—O5^i^	1.680 (4)
V4—O12^i^	1.921 (4)
V4—O2	1.953 (4)
V4—O1^i^	2.097 (4)
V4—O1	2.111 (4)
V5—O13	1.590 (5)
V5—O14	1.835 (4)
V5—O10	1.864 (4)
V5—O3	1.881 (4)
V5—O4	2.045 (4)
V5—O1	2.318 (4)

Symmetry code: (i) 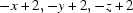.

**Table 2:** Hydrogen-bond geometry (Å, °)

*D*—H⋯*A*	*D*—H	H⋯*A*	*D*⋯*A*	*D*—H⋯*A*
N1—H1*A*⋯O4^ii^	0.86	2.44	3.217 (9)	150
N1—H1*A*⋯O5^iii^	0.86	2.55	3.286 (8)	144
N1—H1*B*⋯O10^iv^	0.86	2.21	3.058 (8)	169
N2—H2*A*⋯O2^ii^	0.86	1.85	2.697 (7)	168
N3—H3*A*⋯O9^iv^	0.86	2.17	2.969 (7)	155
N3—H3*A*⋯O7^v^	0.86	2.48	3.017 (7)	122
N3—H3*B*⋯O14	0.86	2.09	2.908 (7)	158
N4—H4*A*⋯O12^v^	0.86	1.84	2.698 (6)	174
N5—H5*A*⋯O7^v^	0.86	2.23	3.085 (8)	175
N5—H5*B*⋯O9	0.86	2.22	3.057 (8)	164
N6—H6⋯O8^v^	0.86	1.87	2.709 (7)	166
O15—H15*A*⋯O6^vi^	0.85	2.09	2.926 (8)	166
O15—H15*B*⋯O7^ii^	0.85	2.13	2.977 (8)	180

Symmetry codes: (ii) 
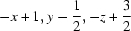
; (iii) 
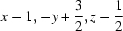
; (iv) 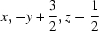; (v) 
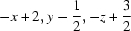
; (vi) 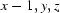.

**Fig. 1.**
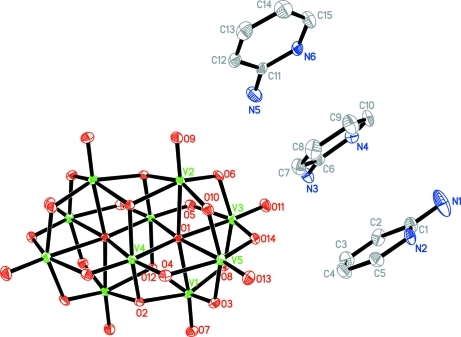
A view of the structure of (I) with displacement ellipsoids drawn at the 30% probability level; H atoms and the water molecule are omitted for clarity.

**Fig. 2.**
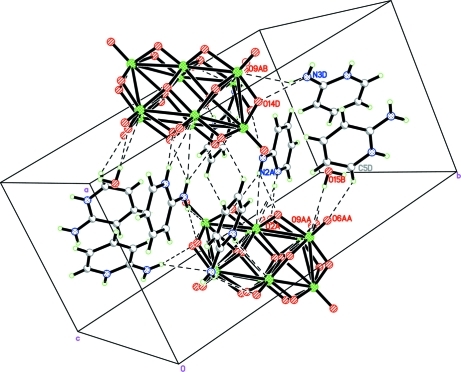
The packing in (I), with hydrogen bonds indicated by dashed lines.

## supplementary crystallographic information

### Comment

Oxovanadates and peroxovanadium compounds are of great interest in biochemistry and medicine because of their diverse biological activites (Pacigová *et al.*, 2007). Of them, decavanadates have showm high affinity for selected kinases and phosphorylase and has been used to facilitate crystallization of proteins. Herein, we report the structure of the title compound, (I), containing decavanadate anions, 2-aminopyridinium cations and water molecules (Fig. 1).

Compound (I) consists of a centrosymmetric [V_10_O_28_]^6-^ polyanion, two distinct 2-aminopyridium cations and a water molecule (Fig. 1). The [V_10_O_28_]^6-^ unit is constructed by ten VO_6_ octahedra connected with each other *via* edge-sharing oxygen atoms.  The different coordination of the oxygen atoms in the molecule results in different V—O bond distances (Table 1). The V—O (one coordinated oxygen) double bond distances range from 1.590 (5) to 1.616 (4) Å; the V—O (two coordinated oxygen) single bond distances range from 1.676 (4) to 2.045 (4) Å; the V—O (three coordinated oxygen) single bond distances range from 1.921 (4) to 2.038 (4)Å and the V—O (six coordinated oxygen) single bond distances are even longer (2.097 (4) to 2.318 (4) Å). These V—O bond types are similar to those in related compounds (Gong *et al.*, 2006).

A three-dimensional supramolecular hydrogen-bonding network is observed in the crystal structure of (I); details are given in Table 2.  All the (C_5_H_7_N_2_)^+^ cations and water molecules are involved in hydrogen bonds with either terminal or bridging O atoms in the [V_10_O_28_]^6-^ anion (Fig. 2).

### Experimental

A hot aqueous VOSO_4_ solution (1 mmol) was added dropwise to a stirred solution of 2-aminopyridine (1 mmol), which was dissolved in 20 ml of ethanol and refluxed for 4 h. Then the filtrate was kept open to slowly evaporate for a few days, depositiong yellow blocks of (I).

### Refinement

The H atoms were placed in geometrically idealized positions (C—H = 0.93Å, N—H =0.86Å, O—H = 0.85Å) and refined as riding with *U*_iso_(H) = 1.2*U*_eq_(carrier).

### Crystal data

**Table:**

(C_5_H_7_N_2_)_6_[V_10_O_28_]·2H_2_O	*F*(000) = 1560
*M**_r_* = 1564.19	*D*_x_ = 2.048 Mg m^−^^3^
Monoclinic, *P*2_1_/*c*	Mo *K*α radiation, λ = 0.71073 Å
Hall symbol: -P 2ybc	Cell parameters from 3889 reflections
*a* = 9.840 (3) Å	θ = 2.2–27.0°
*b* = 18.180 (6) Å	µ = 1.86 mm^−^^1^
*c* = 14.299 (5) Å	*T* = 298 K
β = 97.416 (4)°	Block, yellow
*V* = 2536.6 (14) Å^3^	0.40 × 0.20 × 0.20 mm
*Z* = 2	

### Data collection

**Table:**

Bruker SMART 1K CCD diffractometer	4341 independent reflections
Radiation source: fine-focus sealed tube	3949 reflections with *I* > 2σ(*I*)
graphite	*R*_int_ = 0.042
ω scans	θ_max_ = 25.0°, θ_min_ = 2.9°
Absorption correction: multi-scan (*SADABS*; Bruker, 2000)	*h* = −11→11
*T*_min_ = 0.523, *T*_max_ = 0.707	*k* = −21→18
9858 measured reflections	*l* = −16→12

### Refinement

**Table:**

Refinement on *F*^2^	Primary atom site location: structure-invariant direct methods
Least-squares matrix: full	Secondary atom site location: difference Fourier map
*R*[*F*^2^ > 2σ(*F*^2^)] = 0.074	Hydrogen site location: inferred from neighbouring sites
*wR*(*F*^2^) = 0.128	H-atom parameters constrained
*S* = 1.29	*w* = 1/[σ^2^(*F*_o_^2^) + (0.0095*P*)^2^ + 8.6635*P*] where *P* = (*F*_o_^2^ + 2*F*_c_^2^)/3
4341 reflections	(Δ/σ)_max_ = 0.002
370 parameters	Δρ_max_ = 0.53 e Å^−^^3^
0 restraints	Δρ_min_ = −0.45 e Å^−^^3^

### Special details

**Table:**

Geometry. All e.s.d.'s (except the e.s.d. in the dihedral angle between two l.s. planes) are estimated using the full covariance matrix. The cell e.s.d.'s are taken into account individually in the estimation of e.s.d.'s in distances, angles and torsion angles; correlations between e.s.d.'s in cell parameters are only used when they are defined by crystal symmetry. An approximate (isotropic) treatment of cell e.s.d.'s is used for estimating e.s.d.'s involving l.s. planes.
Refinement. Refinement of *F*^2^ against ALL reflections. The weighted *R*-factor *wR* and goodness of fit *S* are based on *F*^2^, conventional *R*-factors *R* are based on *F*, with *F* set to zero for negative *F*^2^. The threshold expression of *F*^2^ > σ(*F*^2^) is used only for calculating *R*-factors(gt) *etc*. and is not relevant to the choice of reflections for refinement. *R*-factors based on *F*^2^ are statistically about twice as large as those based on *F*, and *R*- factors based on ALL data will be even larger.

### Fractional atomic coordinates and isotropic or equivalent isotropic displacement parameters (Å^2^)

**Table:**

	*x*	*y*	*z*	*U*_iso_*/*U*_eq_	
V1	0.92911 (10)	1.06195 (6)	0.82727 (7)	0.0219 (3)	
V2	0.93856 (11)	0.85376 (6)	0.99424 (7)	0.0223 (3)	
V3	1.01726 (11)	0.90023 (6)	0.79935 (8)	0.0261 (3)	
V4	0.84711 (10)	1.01767 (6)	1.02703 (7)	0.0207 (3)	
V5	0.72947 (11)	0.93367 (6)	0.84923 (8)	0.0248 (3)	
O1	0.9456 (4)	0.9637 (2)	0.9237 (3)	0.0187 (9)	
O2	0.8818 (4)	1.1018 (2)	0.9486 (3)	0.0200 (9)	
O3	0.7620 (4)	1.0262 (2)	0.7977 (3)	0.0260 (10)	
O4	0.6972 (4)	0.9938 (2)	0.9652 (3)	0.0257 (10)	
O5	1.1928 (4)	0.9330 (2)	0.8810 (3)	0.0262 (10)	
O6	1.0166 (4)	0.8267 (2)	0.8929 (3)	0.0258 (10)	
O7	0.9299 (4)	1.1366 (2)	0.7659 (3)	0.0302 (10)	
O8	1.0138 (4)	0.9970 (2)	0.7546 (3)	0.0244 (10)	
O9	0.9418 (5)	0.7791 (2)	1.0547 (3)	0.0307 (11)	
O10	0.7627 (4)	0.8570 (2)	0.9357 (3)	0.0263 (10)	
O11	1.0796 (5)	0.8564 (3)	0.7182 (3)	0.0357 (11)	
O12	1.1132 (4)	1.0743 (2)	0.9091 (3)	0.0196 (9)	
O13	0.5753 (5)	0.9180 (3)	0.8055 (3)	0.0356 (11)	
O14	0.8326 (4)	0.8887 (2)	0.7676 (3)	0.0257 (10)	
N1	0.4744 (8)	0.6382 (4)	0.4937 (6)	0.075 (3)	
H1A	0.4151	0.6036	0.4825	0.090*	
H1B	0.5513	0.6357	0.4713	0.090*	
N2	0.3280 (5)	0.6980 (3)	0.5806 (4)	0.0319 (13)	
H2A	0.2685	0.6638	0.5665	0.038*	
N3	0.8180 (6)	0.7312 (3)	0.7329 (4)	0.0397 (15)	
H3A	0.8710	0.7189	0.6922	0.048*	
H3B	0.8259	0.7738	0.7591	0.048*	
N4	0.7146 (5)	0.6182 (3)	0.7122 (4)	0.0301 (13)	
H4A	0.7710	0.6077	0.6730	0.036*	
C1	0.4480 (7)	0.6944 (4)	0.5460 (5)	0.0361 (17)	
C2	0.5431 (7)	0.7514 (4)	0.5687 (5)	0.0387 (18)	
H2	0.6274	0.7507	0.5459	0.046*	
C3	0.5103 (8)	0.8073 (4)	0.6245 (6)	0.0442 (19)	
H3	0.5722	0.8456	0.6388	0.053*	
C4	0.3861 (7)	0.8082 (4)	0.6606 (6)	0.0424 (19)	
H4	0.3651	0.8458	0.7003	0.051*	
C5	0.2971 (7)	0.7535 (4)	0.6369 (5)	0.0355 (17)	
H5	0.2127	0.7538	0.6597	0.043*	
C6	0.7244 (7)	0.6848 (4)	0.7549 (5)	0.0291 (15)	
C7	0.6326 (7)	0.7000 (4)	0.8184 (5)	0.0418 (19)	
H7	0.6362	0.7453	0.8489	0.050*	
C8	0.5373 (7)	0.6501 (5)	0.8361 (5)	0.045 (2)	
H8	0.4777	0.6604	0.8798	0.054*	
C9	0.5284 (8)	0.5829 (5)	0.7885 (6)	0.048 (2)	
H9	0.4607	0.5490	0.7979	0.057*	
C10	0.6201 (7)	0.5680 (4)	0.7286 (5)	0.0387 (18)	
H10	0.6179	0.5226	0.6983	0.046*	
N5	0.9609 (7)	0.6459 (4)	0.9265 (5)	0.0518 (18)	
H5A	0.9958	0.6421	0.8746	0.062*	
H5B	0.9708	0.6859	0.9587	0.062*	
N6	0.8778 (6)	0.5280 (3)	0.9057 (4)	0.0324 (13)	
H6	0.9138	0.5261	0.8540	0.039*	
C11	0.8923 (7)	0.5903 (4)	0.9568 (5)	0.0301 (15)	
C12	0.8313 (8)	0.5922 (4)	1.0402 (5)	0.0388 (18)	
H12	0.8382	0.6344	1.0773	0.047*	
C13	0.7629 (8)	0.5333 (5)	1.0668 (5)	0.049 (2)	
H13	0.7232	0.5351	1.1224	0.058*	
C14	0.7509 (9)	0.4700 (5)	1.0124 (6)	0.052 (2)	
H14	0.7034	0.4293	1.0305	0.062*	
C15	0.8107 (8)	0.4690 (4)	0.9313 (6)	0.046 (2)	
H15	0.8048	0.4270	0.8939	0.055*	
O15	0.2400 (7)	0.7219 (4)	0.8838 (5)	0.083 (2)	
H15A	0.1820	0.7567	0.8795	0.125*	
H15B	0.1917	0.6976	0.8409	0.125*	

### Atomic displacement parameters (Å^2^)

**Table:**

	*U*^11^	*U*^22^	*U*^33^	*U*^12^	*U*^13^	*U*^23^
V1	0.0254 (6)	0.0199 (6)	0.0200 (6)	−0.0005 (4)	0.0013 (4)	0.0038 (4)
V2	0.0268 (6)	0.0164 (5)	0.0237 (6)	−0.0012 (4)	0.0038 (5)	0.0001 (4)
V3	0.0308 (6)	0.0240 (6)	0.0242 (6)	−0.0007 (5)	0.0068 (5)	−0.0047 (5)
V4	0.0211 (5)	0.0193 (5)	0.0223 (6)	0.0022 (4)	0.0047 (4)	0.0013 (4)
V5	0.0229 (6)	0.0272 (6)	0.0238 (6)	−0.0033 (5)	0.0008 (5)	−0.0018 (5)
O1	0.019 (2)	0.018 (2)	0.020 (2)	−0.0005 (16)	0.0022 (17)	0.0005 (17)
O2	0.022 (2)	0.018 (2)	0.021 (2)	0.0030 (17)	0.0057 (17)	0.0021 (17)
O3	0.025 (2)	0.028 (2)	0.024 (2)	0.0028 (18)	−0.0015 (19)	0.0031 (19)
O4	0.019 (2)	0.028 (2)	0.031 (3)	0.0003 (18)	0.0050 (18)	0.0031 (19)
O5	0.023 (2)	0.027 (2)	0.030 (3)	0.0042 (18)	0.0085 (19)	−0.0017 (19)
O6	0.034 (2)	0.016 (2)	0.029 (3)	0.0033 (18)	0.008 (2)	−0.0041 (18)
O7	0.032 (2)	0.029 (3)	0.030 (3)	−0.001 (2)	0.003 (2)	0.008 (2)
O8	0.032 (2)	0.024 (2)	0.018 (2)	−0.0013 (19)	0.0040 (18)	−0.0012 (18)
O9	0.044 (3)	0.022 (2)	0.027 (3)	−0.005 (2)	0.006 (2)	0.0011 (19)
O10	0.030 (2)	0.022 (2)	0.028 (3)	−0.0047 (19)	0.0069 (19)	−0.0013 (19)
O11	0.048 (3)	0.033 (3)	0.028 (3)	−0.001 (2)	0.014 (2)	−0.005 (2)
O12	0.024 (2)	0.017 (2)	0.019 (2)	−0.0020 (17)	0.0035 (17)	0.0010 (17)
O13	0.031 (3)	0.040 (3)	0.033 (3)	0.000 (2)	−0.006 (2)	−0.003 (2)
O14	0.032 (2)	0.021 (2)	0.023 (2)	−0.0006 (19)	0.0014 (19)	−0.0029 (18)
N1	0.053 (4)	0.072 (6)	0.109 (7)	−0.026 (4)	0.043 (4)	−0.056 (5)
N2	0.026 (3)	0.032 (3)	0.038 (4)	−0.011 (2)	0.005 (3)	−0.013 (3)
N3	0.040 (3)	0.034 (3)	0.047 (4)	−0.002 (3)	0.013 (3)	−0.013 (3)
N4	0.025 (3)	0.036 (3)	0.032 (3)	0.002 (2)	0.011 (2)	−0.002 (3)
C1	0.033 (4)	0.040 (4)	0.037 (4)	−0.005 (3)	0.008 (3)	−0.009 (3)
C2	0.032 (4)	0.049 (5)	0.036 (4)	−0.018 (3)	0.008 (3)	0.002 (4)
C3	0.039 (4)	0.033 (4)	0.058 (5)	−0.017 (3)	−0.003 (4)	−0.001 (4)
C4	0.037 (4)	0.031 (4)	0.057 (5)	0.001 (3)	0.000 (4)	−0.012 (4)
C5	0.035 (4)	0.031 (4)	0.041 (4)	−0.002 (3)	0.006 (3)	−0.002 (3)
C6	0.027 (3)	0.033 (4)	0.027 (4)	0.005 (3)	−0.001 (3)	0.000 (3)
C7	0.036 (4)	0.048 (5)	0.042 (5)	0.012 (4)	0.010 (4)	−0.014 (4)
C8	0.030 (4)	0.067 (6)	0.042 (5)	0.001 (4)	0.019 (3)	−0.010 (4)
C9	0.040 (4)	0.049 (5)	0.057 (5)	−0.013 (4)	0.015 (4)	−0.010 (4)
C10	0.045 (4)	0.032 (4)	0.038 (4)	−0.008 (3)	0.003 (4)	−0.014 (3)
N5	0.068 (5)	0.035 (4)	0.054 (5)	−0.001 (3)	0.016 (4)	−0.005 (3)
N6	0.039 (3)	0.037 (3)	0.022 (3)	0.008 (3)	0.008 (3)	−0.008 (3)
C11	0.027 (3)	0.037 (4)	0.024 (4)	0.011 (3)	−0.008 (3)	−0.005 (3)
C12	0.049 (5)	0.044 (5)	0.024 (4)	0.016 (4)	0.008 (3)	−0.006 (3)
C13	0.051 (5)	0.066 (6)	0.033 (5)	0.007 (4)	0.019 (4)	−0.001 (4)
C14	0.056 (5)	0.051 (5)	0.052 (5)	−0.009 (4)	0.021 (4)	0.003 (4)
C15	0.056 (5)	0.036 (4)	0.047 (5)	−0.004 (4)	0.009 (4)	−0.014 (4)
O15	0.062 (4)	0.098 (6)	0.086 (5)	0.022 (4)	−0.004 (4)	−0.013 (4)

### Geometric parameters (Å, °)

**Table:**

V1—O7	1.616 (4)	N2—H2A	0.8600
V1—O3	1.767 (4)	N3—C6	1.316 (9)
V1—O8	1.842 (4)	N3—H3A	0.8600
V1—O2	1.991 (4)	N3—H3B	0.8599
V1—O12	2.038 (4)	N4—C10	1.345 (9)
V1—O1	2.250 (4)	N4—C6	1.353 (8)
V2—O9	1.608 (4)	N4—H4A	0.8599
V2—O6	1.794 (4)	C1—C2	1.405 (9)
V2—O10	1.823 (4)	C2—C3	1.357 (11)
V2—O12^i^	2.015 (4)	C2—H2	0.9299
V2—O2^i^	2.017 (4)	C3—C4	1.387 (11)
V2—O1	2.243 (4)	C3—H3	0.9300
V3—O11	1.594 (4)	C4—C5	1.339 (10)
V3—O14	1.827 (4)	C4—H4	0.9300
V3—O8	1.871 (4)	C5—H5	0.9300
V3—O6	1.891 (4)	C6—C7	1.389 (9)
V3—O5	2.045 (4)	C7—C8	1.352 (11)
V3—O1	2.305 (4)	C7—H7	0.9300
V4—O4	1.676 (4)	C8—C9	1.396 (11)
V4—O5^i^	1.680 (4)	C8—H8	0.9300
V4—O12^i^	1.921 (4)	C9—C10	1.349 (10)
V4—O2	1.953 (4)	C9—H9	0.9300
V4—O1^i^	2.097 (4)	C10—H10	0.9300
V4—O1	2.111 (4)	N5—C11	1.319 (9)
V5—O13	1.590 (5)	N5—H5A	0.8600
V5—O14	1.835 (4)	N5—H5B	0.8601
V5—O10	1.864 (4)	N6—C15	1.335 (9)
V5—O3	1.881 (4)	N6—C11	1.346 (8)
V5—O4	2.045 (4)	N6—H6	0.8600
V5—O1	2.318 (4)	C11—C12	1.403 (9)
O1—V4^i^	2.097 (4)	C12—C13	1.346 (11)
O2—V2^i^	2.017 (4)	C12—H12	0.9300
O5—V4^i^	1.680 (4)	C13—C14	1.386 (11)
O12—V4^i^	1.921 (4)	C13—H13	0.9300
O12—V2^i^	2.015 (4)	C14—C15	1.366 (11)
N1—C1	1.312 (9)	C14—H14	0.9300
N1—H1A	0.8599	C15—H15	0.9300
N1—H1B	0.8600	O15—H15A	0.8499
N2—C1	1.339 (8)	O15—H15B	0.8496
N2—C5	1.350 (8)		
			
O7—V1—O3	104.4 (2)	V1—O1—V3	85.95 (14)
O7—V1—O8	101.4 (2)	V4^i^—O1—V5	170.8 (2)
O3—V1—O8	95.96 (19)	V4—O1—V5	87.36 (14)
O7—V1—O2	100.7 (2)	V2—O1—V5	85.25 (13)
O3—V1—O2	91.58 (18)	V1—O1—V5	85.16 (14)
O8—V1—O2	154.05 (18)	V3—O1—V5	83.19 (13)
O7—V1—O12	98.8 (2)	V4—O2—V1	107.04 (19)
O3—V1—O12	155.27 (18)	V4—O2—V2^i^	106.87 (19)
O8—V1—O12	87.81 (17)	V1—O2—V2^i^	101.98 (17)
O2—V1—O12	75.49 (16)	V1—O3—V5	115.8 (2)
O7—V1—O1	173.8 (2)	V4—O4—V5	110.3 (2)
O3—V1—O1	81.16 (17)	V4^i^—O5—V3	109.7 (2)
O8—V1—O1	80.48 (16)	V2—O6—V3	114.8 (2)
O2—V1—O1	76.19 (15)	V1—O8—V3	113.5 (2)
O12—V1—O1	75.36 (15)	V2—O10—V5	113.8 (2)
O9—V2—O6	102.8 (2)	V4^i^—O12—V2^i^	107.03 (18)
O9—V2—O10	103.1 (2)	V4^i^—O12—V1	106.72 (18)
O6—V2—O10	96.7 (2)	V2^i^—O12—V1	100.42 (17)
O9—V2—O12^i^	99.71 (19)	V3—O14—V5	113.9 (2)
O6—V2—O12^i^	154.72 (18)	C1—N1—H1A	119.8
O10—V2—O12^i^	89.15 (18)	C1—N1—H1B	120.1
O9—V2—O2^i^	99.5 (2)	H1A—N1—H1B	120.0
O6—V2—O2^i^	89.59 (18)	C1—N2—C5	122.3 (6)
O10—V2—O2^i^	154.54 (18)	C1—N2—H2A	119.0
O12^i^—V2—O2^i^	75.43 (16)	C5—N2—H2A	118.7
O9—V2—O1	173.9 (2)	C6—N3—H3A	120.0
O6—V2—O1	80.77 (17)	C6—N3—H3B	120.0
O10—V2—O1	81.23 (16)	H3A—N3—H3B	120.0
O12^i^—V2—O1	75.85 (15)	C10—N4—C6	122.6 (6)
O2^i^—V2—O1	75.47 (15)	C10—N4—H4A	118.8
O11—V3—O14	103.0 (2)	C6—N4—H4A	118.6
O11—V3—O8	102.3 (2)	N1—C1—N2	119.8 (7)
O14—V3—O8	92.79 (19)	N1—C1—C2	122.0 (7)
O11—V3—O6	101.5 (2)	N2—C1—C2	118.1 (6)
O14—V3—O6	90.05 (19)	C3—C2—C1	119.1 (7)
O8—V3—O6	154.81 (18)	C3—C2—H2	120.5
O11—V3—O5	100.7 (2)	C1—C2—H2	120.4
O14—V3—O5	156.20 (18)	C2—C3—C4	121.0 (7)
O8—V3—O5	84.18 (18)	C2—C3—H3	119.4
O6—V3—O5	83.21 (18)	C4—C3—H3	119.6
O11—V3—O1	175.1 (2)	C5—C4—C3	118.5 (7)
O14—V3—O1	81.71 (16)	C5—C4—H4	120.8
O8—V3—O1	78.46 (16)	C3—C4—H4	120.8
O6—V3—O1	77.20 (16)	C4—C5—N2	121.0 (7)
O5—V3—O1	74.55 (15)	C4—C5—H5	119.5
O4—V4—O5^i^	105.8 (2)	N2—C5—H5	119.5
O4—V4—O12^i^	97.83 (19)	N3—C6—N4	118.4 (6)
O5^i^—V4—O12^i^	98.45 (19)	N3—C6—C7	124.3 (7)
O4—V4—O2	96.17 (19)	N4—C6—C7	117.3 (6)
O5^i^—V4—O2	96.12 (19)	C8—C7—C6	120.9 (7)
O12^i^—V4—O2	156.20 (17)	C8—C7—H7	119.4
O4—V4—O1^i^	166.03 (18)	C6—C7—H7	119.7
O5^i^—V4—O1^i^	88.08 (18)	C7—C8—C9	119.9 (7)
O12^i^—V4—O1^i^	81.50 (16)	C7—C8—H8	120.3
O2—V4—O1^i^	80.29 (16)	C9—C8—H8	119.9
O4—V4—O1	87.89 (18)	C10—C9—C8	118.7 (7)
O5^i^—V4—O1	166.22 (19)	C10—C9—H9	120.7
O12^i^—V4—O1	81.02 (16)	C8—C9—H9	120.6
O2—V4—O1	80.36 (16)	N4—C10—C9	120.5 (7)
O1^i^—V4—O1	78.21 (17)	N4—C10—H10	119.7
O13—V5—O14	104.4 (2)	C9—C10—H10	119.7
O13—V5—O10	101.8 (2)	C11—N5—H5A	120.0
O14—V5—O10	91.43 (19)	C11—N5—H5B	120.0
O13—V5—O3	102.0 (2)	H5A—N5—H5B	120.0
O14—V5—O3	90.73 (19)	C15—N6—C11	123.3 (6)
O10—V5—O3	154.79 (19)	C15—N6—H6	118.4
O13—V5—O4	100.0 (2)	C11—N6—H6	118.3
O14—V5—O4	155.60 (18)	N5—C11—N6	119.3 (6)
O10—V5—O4	83.94 (18)	N5—C11—C12	123.6 (7)
O3—V5—O4	83.79 (18)	N6—C11—C12	117.1 (7)
O13—V5—O1	174.4 (2)	C13—C12—C11	120.4 (7)
O14—V5—O1	81.21 (17)	C13—C12—H12	119.8
O10—V5—O1	78.41 (16)	C11—C12—H12	119.8
O3—V5—O1	77.12 (16)	C12—C13—C14	120.8 (7)
O4—V5—O1	74.39 (15)	C12—C13—H13	119.6
V4^i^—O1—V4	101.79 (16)	C14—C13—H13	119.6
V4^i^—O1—V2	94.50 (15)	C15—C14—C13	118.2 (8)
V4—O1—V2	93.22 (15)	C15—C14—H14	120.9
V4^i^—O1—V1	93.87 (15)	C13—C14—H14	120.9
V4—O1—V1	93.23 (15)	N6—C15—C14	120.3 (7)
V2—O1—V1	168.2 (2)	N6—C15—H15	119.9
V4^i^—O1—V3	87.65 (14)	C14—C15—H15	119.9
V4—O1—V3	170.6 (2)	H15A—O15—H15B	91.4
V2—O1—V3	86.07 (14)		

Symmetry codes: (i) −*x*+2, −*y*+2, −*z*+2.

### Hydrogen-bond geometry (Å, °)

**Table:**

*D*—H···*A*	*D*—H	H···*A*	*D*···*A*	*D*—H···*A*
N1—H1A···O4^ii^	0.86	2.44	3.217 (9)	150
N1—H1A···O5^iii^	0.86	2.55	3.286 (8)	144
N1—H1B···O10^iv^	0.86	2.21	3.058 (8)	169
N2—H2A···O2^ii^	0.86	1.85	2.697 (7)	168
N3—H3A···O9^iv^	0.86	2.17	2.969 (7)	155
N3—H3A···O7^v^	0.86	2.48	3.017 (7)	122
N3—H3B···O14	0.86	2.09	2.908 (7)	158
N4—H4A···O12^v^	0.86	1.84	2.698 (6)	174
N5—H5A···O7^v^	0.86	2.23	3.085 (8)	175
N5—H5B···O9	0.86	2.22	3.057 (8)	164
N6—H6···O8^v^	0.86	1.87	2.709 (7)	166
O15—H15A···O6^vi^	0.85	2.09	2.926 (8)	166
O15—H15B···O7^ii^	0.85	2.13	2.977 (8)	180

Symmetry codes: (ii) −*x*+1, *y*−1/2, −*z*+3/2; (iii) *x*−1, −*y*+3/2, *z*−1/2; (iv) *x*, −*y*+3/2, *z*−1/2; (v) −*x*+2, *y*−1/2, −*z*+3/2; (vi) *x*−1, *y*, *z*.

## References

[bb1] Bruker (2000). *SMART*, *SAINT* and *SADABS* Bruker AXS Inc., Madison, Wisconsin, USA.

[bb2] Gong, Y., Hu, C., Li, H., Tang, W., Huang, K. & Hou, W. B. (2006). *J. Mol. Struct.* **784**, 228–238.

[bb3] Pacigová, S., Rakovský, E., Sivák, M. & Žák, Z. (2007). *Acta Cryst.* C**63**, m419–m422. 10.1107/S0108270107028557 17762113

[bb4] Sheldrick, G. M. (2008). *Acta Cryst.* A**64**, 112–122. 10.1107/S0108767307043930 18156677

